# Use of beta-blockers and depressive symptoms in hypertensive older adults: a multicenter study

**DOI:** 10.47487/apcyccv.v6i3.520

**Published:** 2025-09-24

**Authors:** Mayra Valdivia-Herrera, P. Alejandra Goicochea-Romero, Christopher Butler Vallejo, Ian Falvy-Bockos, Carmen Eliana Peralta Vargas, Fernando Runzer-Colmenares

**Affiliations:** 1 . CHANGE Research Working Group, Carrera de Medicina Humana, Universidad Científica del Sur, Lima, Peru. Universidad Científica del Sur CHANGE Research Working Group Carrera de Medicina Humana Universidad Científica del Sur Lima Peru; 2 . Centro Médico Naval del Perú, Lima, Peru. Centro Médico Naval del Perú Lima Peru; 3 . Hospital Central FAP del Perú, Lima, Peru. Hospital Central FAP del Perú Lima Peru

**Keywords:** Aged, Hypertension, Adrenergic beta-Antagonists, Depression, Peru, Anciano, Hipertensión, Antagonistas Adrenérgicos beta, Depresión, Perú

## Abstract

**Objectives.:**

To evaluate the association between beta-blocker use and the presence of depressive symptoms in older adults with hypertension (HTN).

**Materials and Methods.:**

A cross-sectional observational study was conducted among patients from the Central FAP Hospital and the Naval Medical Center. Older adults with a diagnosis of HTN who had been receiving antihypertensive treatment for at least one month were included. Depressive symptoms were assessed using the PHQ-9 questionnaire. Social, clinical, and functional variables were analyzed using bivariate tests (Chi-square and Fisher’s exact test), as well as Poisson regression with robust variance to estimate crude and adjusted prevalence ratios (PR).

**Results.:**

A total of 149 older adults were included. Of these, 27.5% were taking beta-blockers, and 39% presented depressive symptoms. No significant association was found between beta-blocker use and the presence of depressive symptoms (PR:1.09; 95% CI: 0.70-1.69; p = 0.693). In contrast, a higher risk of depressive symptoms was observed among patients with greater frailty (PR: 5.72; 95% CI: 2.17-15.0; p < 0.001) and a lower risk among those with technical or university education (PR: 0.43; 95% CI: 0.25-0.74; p = 0.003).

**Conclusions.:**

No association was found between beta-blocker use and depressive symptoms in patients with HTN. Factors such as frailty, educational level, and duration of HTN diagnosis showed significant associations and should be considered in the comprehensive assessment of emotional risk in this population.

## RESUMEN

**Objetivos.:**

Evaluar la asociación entre el uso de betabloqueadores (BB) y la presencia de síntomas depresivos en adultos mayores con hipertensión arterial (HTA).

**Materiales y métodos.:**

Se realizó un estudio observacional de corte transversal en pacientes del Hospital Central FAP y del Centro Médico Naval. Se incluyeron adultos mayores con diagnóstico de HTA en tratamiento antihipertensivo durante al menos un mes. Los síntomas depresivos se evaluaron mediante el cuestionario PHQ-9. Se analizaron variables sociales, clínicas y funcionales mediante pruebas bivariadas (chi cuadrado y prueba exacta de Fisher), así como regresión de Poisson con varianza robusta para estimar razones de prevalencia (RP) crudas y ajustadas.

**Resultados.:**

Se incluyeron 149 adultos mayores. El 27,5% consumía BB y el 39% presentó síntomas depresivos. No se encontró asociación significativa entre el uso de BB y la presencia de síntomas depresivos (RP: 1,09; IC 95%: 0,70-1,69; p = 0,693). En cambio, se observó mayor riesgo de síntomas depresivos en pacientes con mayor fragilidad (RP: 5,72; IC 95%: 2,17-15,0; p < 0,001) y menor riesgo en aquellos con estudios técnicos o universitarios (RP: 0,43; IC 95%: 0,25-0,74; p = 0,003).

**Conclusiones.:**

No se encontró asociación entre el uso de BB y los síntomas depresivos en pacientes con HTA. Factores como la fragilidad, el nivel educativo y el tiempo de diagnóstico de HTA mostraron asociaciones significativas y deben considerarse en la evaluación integral del riesgo emocional en esta población

## Introduction

Hypertension (HTN) is one of the most prevalent chronic diseases worldwide, affecting more than 1.28 billion people in 2023. Prevalence increases with age, reaching 27% in individuals younger than 60 years and 74% in those older than 80 years. ^1^^,^^2^ In Latin America, HTN is estimated to cause 1.6 million deaths annually and is the leading risk factor for coronary heart disease and stroke. ^3^ In Peru, around 5.5 million people are affected, according to the 2022 Demographic and Family Health Survey (ENDES). ^4^ HTN has also been linked to high levels of stress and negative emotions, with major depression being one of the most frequent comorbid conditions. ^5^

In 2023, major depression was estimated to affect about 280 million people worldwide and to cause more than 700,000 deaths annually from suicide, highlighting its importance as a global public health challenge. ^6^ The disorder is characterised by persistent low mood, hopelessness, sadness, anhedonia, fatigue, and loss of energy, leading to poor quality of life, disability, and impaired social and family functioning. ^6^ Older adults are particularly affected, with a higher prevalence of depressive symptoms and increased mortality ^7^, partly explained by degenerative changes in neurotransmitter metabolism. ^8^ In Peru, the 2020 ENDES survey reported a prevalence of depressive symptoms of 10.8% among older adults. ^9^ Furthermore, a 2018 report from the Pan American Health Organization showed that 8.6% of the Peruvian population lives with disability attributable to major depression, ranking third in the Americas. ^10^

Several studies have examined the association between HTN and major depression, focusing on shared risk factors, treatment adherence, and common pathophysiological pathways. ^5^ Evidence suggests that beta-blockers (BBs), particularly lipophilic and non-selective agents, may increase the prevalence of depressive symptoms owing to the widespread distribution of adrenergic receptors and their ability to cross the blood-brain barrier, resulting in central nervous system effects such as mood changes and sleep disturbances. ^11^

Although the mechanisms of action of these drugs and their central nervous system effects are well described, evidence in older populations remains inconsistent. Boal *et al.* reported that BB use increases the risk of major depression, whereas Tao *et al.* found no such association. ^12^^,^^13^ We therefore aimed to investigate the association between BB use and the presence of depressive symptoms in patients with HTN.

## Materials and methods

### Study design

We conducted an analytical cross-sectional study in older adults with a diagnosis of HTN.

### Study setting

The study was carried out between May and July 2022 in two military health institutions in Peru: the Central Hospital of the Peruvian Air Force (FAP) and the Naval Medical Centre “Cirujano Mayor Santiago Távara”. Data collection took place in outpatient geriatric clinics. Both hospitals provide care for active and retired military personnel and their families.

### Participants

The study population comprised adults aged 60 years and older with a clinical record of HTN and under antihypertensive treatment for at least one month. Patients with moderate-to-severe dementia, severe hearing impairment, antihypertensive use for conditions other than HTN, or ongoing hospitalisation were excluded.

### Sample size and selection

Convenience sampling was used during regular clinic hours, applying predefined eligibility criteria. Sample size was calculated with OpenEpi version 3.01, assuming a 95% confidence level, 80% power, an expected prevalence of major depression of 12% in BB users, and an association magnitude of 3.31. ^14^ According to Kelsey’s formula, 146 participants were required; 149 were ultimately included.

### Variables

All patients underwent an interview composed of independent questions, questionnaires, and scales, which allowed the collection of demographic, clinical, and functional information.

The main exposure variable was BB use, categorised as “yes” (BB use) and “no” (use of any other antihypertensive drug different from BBs). The primary outcome was the presence of depressive symptoms, screened with the ICOPE detection guide, validated and proposed by the World Health Organization (WHO) for comprehensive assessment of older adults. ^15^ In cases with a positive screening result, this was confirmed using the Patient Health Questionnaire-9 (PHQ-9), validated in Latin American older adults, and categorised as: no symptoms (0-5), mild (6-8), moderate (9-14), and moderately severe or severe (15-27). ^16^

Sociodemographic variables included: age in completed years (categorised as 60-69, 70-79, 80-89, and ≥90); biological sex (male/female); marital status at the time of the survey (single, married, widowed, or divorced); educational attainment (highest level of education achieved, stratified as primary, secondary, or technical/university); living arrangement (living alone or accompanied); recent bereavement of a close relative (within the last year, between 1 and 2 years, or more than 2 years); and harmful lifestyle habits (alcohol or cigarette consumption).

Clinical variables included: duration of HTN diagnosis (in years since initial diagnosis, categorised as <10, 10-19, and ≥20 years); type of antihypertensive drug (Angiotensin-Converting Enzyme [ACE] inhibitors, Angiotensin II Receptor Blockers [ARBs], calcium channel blockers, diuretics, and BBs); and number of antihypertensive agents (1, 2, or ≥3). Polypharmacy was also assessed, defined as the number of daily medications consumed excluding antihypertensives, and categorised as 1-2, 3-4, and ≥5. Information on these variables was collected directly from patient interviews and, in some cases (drugs, comorbidities), corroborated through clinical records.

Comorbidities were assessed using the Charlson Comorbidity Index (CCI) ^17^, widely validated in older adults. Frailty was measured using the FRAIL scale, validated in diverse contexts with high diagnostic performance, classifying individuals as robust (0 points), pre-frail (1-2 points), or frail (3-5 points). ^18^Finally, activities of daily living were assessed using the Barthel Index, validated in multiple geographical regions, showing good inter-observer reliability and functional utility. Functional status was categorised as total independence (score 100), mild dependence (91-99), and moderate dependence (61-90). ^19^ Full versions of the questionnaires and instruments applied (PHQ-9, Barthel Index, FRAIL) are included in the Supplementary Material.

### Statistical analysis

Data were coded in a Microsoft Excel spreadsheet and subsequently exported to Stata version 16.0. The assumption of normality was assessed using the Shapiro-Wilk test. Categorical variables were described as absolute frequencies and percentages, while quantitative variables were expressed as mean and standard deviation according to the Shapiro-Wilk results. In the bivariate analysis to determine the association between BB use and depressive symptoms, the chi-squared test was applied; when any expected frequency was <5, Fisher’s exact test was used. For multivariate analysis, a Poisson regression model with robust variance was employed to estimate crude and adjusted prevalence ratios (PRs). Covariates with p<0.20 in the bivariate analysis, as well as clinically relevant variables documented in the literature (age and sex), were included in the model. Four multivariate models were constructed to assess the robustness of the main association under different adjustment scenarios, including combinations of sociodemographic and clinical variables: model A included sex and age; model B included sex, education level, and duration of HTN diagnosis; model C included sex, education level, and frailty; and model D included sex, education level, and the CCI. This approach allowed verification of the consistency of findings while controlling for potential confounding factors. A p-value <0.05 was considered statistically significant, and 95% confidence intervals (95% CIs) were reported.

### Ethical aspects

All patients provided written informed consent before data collection. The study was approved by the Ethics Committee of Universidad Científica del Sur and by the ethics committees of each participating hospital. The project was funded by the Faculty of Human Medicine, Universidad Científica del Sur (Cabieses Scholarship).

## Results

A total of 156 older adults with HTN were initially considered; seven were excluded (due to severe hearing impairment or refusal to participate), leaving 149 for analysis. The sample selection process is detailed in the Supplementary Material.

Of the 149 participants surveyed during the study period, 54.36% were men. Most were aged 70-79 years, married, living with others, and had attained technical or university education. Thirty-six percent reported a duration of HTN diagnosis of more than 20 years. The most commonly used antihypertensive drugs were ARBs (64·43%), followed by calcium channel blockers (30·87%) and BBs (27·52%). Most patients (54%) were on antihypertensive monotherapy, while polypharmacy (≥5 non-antihypertensive drugs) was present in 46% of cases.

Regarding clinical conditions, 59% of participants were classified as pre-frail and 54% presented with mild dependence according to the Barthel Index. With respect to mental health, according to the PHQ-9, 58 older adults (38·9%) presented with depressive symptoms. Of these, 62% reported mild symptoms, 31% moderate, and 7% moderately severe or severe. Among these 58 cases, 70·7% were receiving antihypertensives other than BBs (Table 1).

**Table 1:** Clinical and sociodemographic characteristics of the study sample (n = 149)

Variables	Total (n = 149) n (%)	Beta-blocker (n = 41) n (%)	Non-beta-blocker (n = 108) n (%)	p-value
Hospital				
Naval	98 (65.8)	24 (58.5)	74 (68.5)	0.251^a^
Air force	51 (34.2)	17 (41.5)	34 (31.5)	
Age				
60-69	21 (14.1)	4 (9.8)	17 (15.7)	0.807^a^
70-79	58 (38.9)	16 (39.0)	42 (38.9)	
80-89	47 (31.6)	14 (34.1)	33 (30.6)	
≥90 years	23 (15.4)	7 (17.1)	16 (14.8)	
Sex				
Female	68 (46.6)	18 (43.9)	50 (46.3)	0.793^a^
Male	81 (54.4)	23 (56.1)	58 (53.7)	
Marital status				
Single	3 (2.0)	1 (2.4)	2 (1.9)	0.227^b^
Married	98 (65.8)	23 (56.1)	75 (69.4)	
Widowed / Divorced	48 (32.2)	17 (41.5)	31 (28.7)	
Educational level				
Primary	43 (28.8)	9 (22.0)	34 (31.8)	0.475^a^
Secondary	46 (30.9)	13 (31.7)	32 (29.9)	
Technical / University	60 (40.3)	19 (46.3)	41 (38.3)	
Living arrangement				
Lives alone	9 (6.0)	3 (7.3)	6 (5.6)	0.687^b^
Lives with others	140 (94.0)	38 (92.7)	102 (94.4)	
Recent loss				
>2 years	91 (61.1)	27 (65.9)	64 (59.3)	0.699^a^
1-2 years	18 (12.1)	5 (12.2)	13 (12.0)	
<1 year	40 (26.8)	9 (21.9)	31 (28.7)	
Harmful habits				
Tobacco	8 (5.4)	3 (7.3)	5 (4.6)	0.516^b^
Alcohol	25 (16.8)	6 (14.6)	19 (17.6)	0.666^a^
Time since HTN diagnosis				
<10 years	47 (31.5)	10 (24.4)	37 (34.3)	0.264^a^
10-19 years	48 (32.2)	12 (29.3)	36 (33.3)	
≥20 years	54 (36.3)	19 (46.3)	35 (32.4)	
Type of antihypertensive*				
ACE inhibitors	38 (25.5)	6 (14.6)	32 (29.6)	0.091ª
ARBs	96 (64.4)	25 (61.0)	71 (65.7)	0.587ª
Calcium channel blockers	46 (30.9)	13 (31.7)	33 (30.6)	0.892ª
Diuretics	24 (16.1)	5 (12.2)	19 (17.6)	0.423ª
Number of antihypertensive drugs						
1	81 (54.4)	7 (17.1)	74 (68.5)	<0.001^a^
2	45 (30.2)	22 (53.6)	23 (21.3)	
3 or more	23 (15.4)	12 (29.3)	11 (10.2)	
Polypharmacy				
1-2	25 (16.8)	1 (2.4)	24 (22.2)	0.001^b^
3-4	55 (36.9)	13 (31.7)	42 (38.9)	
5 or more	69 (46.3)	27 (65.9)	42 (38.9)	
Charlson Comorbidity Index				
0-2	9 (6.0)	1 (2.4)	8 (7.4)	0.096^a^
3-4	66 (44.3)	14 (34.2)	52 (48.2)	
5 or more	74 (49.7)	26 (63.4)	48 (44.4)	
Frailty Index				
Non-frail	35 (23.5)	6 (14.6)	29 (26.9)	0.178^a^
Pre-frail	88 (59.1)	29 (70.8)	59 (54.6)	
Frail	26 (17.4)	6 (14.6)	20 (18.5)	
Barthel Index of functionality				
Independent	65 (43.6)	19 (46.3)	46 (42.6)	0.227^b^
Mild dependency	81 (54.4)	20 (48.8)	61 (56.5)	
Moderate dependency	3 (2.0)	2 (4.9)	1 (0.9)	
PHQ-9				
No depressive symptoms	91 (61.1)	24 (58.5)	67 (62.0)	0.696^a^
Depressive symptoms	58 (38.9)	17 (41.5)	41 (38.0)	

HTN: hypertension. ACE inhibitors: angiotensin-converting enzyme inhibitors. ARBs: angiotensin II receptor blockers. PHQ-9 = Patient Health Questionnaire-9. n: number; %: percentage. * Only categories with drug use are shown. ^a^ Chi-squared test. ^b^ Fisher’s exact test.

In the bivariate analysis (Table 2), no significant differences were found between groups with and without depressive symptoms in terms of sex, age, marital status, living arrangement, bereavement of a close relative, harmful habits, or level of dependence. However, education level showed a significant association (p=0.005), with a lower frequency of depressive symptoms among those with technical or university studies. Associations were also observed with the Charlson Comorbidity Index (p=0.05) and frailty status (p<0.001). By contrast, BB use was not significantly associated with the presence of depressive symptoms (p=0.696).

**Table 2:** Bivariate analysis between depressive symptoms and antihypertensive treatment (n = 149)

Variables	Presence of depressive symptoms n (%)	Absence of depressive symptoms n (%)	p-value
Overall	58 (38.9)	91 (61.1)	
Hospital			
Naval	40 (40.8)	58 (59.2)	0.512^a^
Air force	18 (35.3)	33 (64.7)	
Age			
60-69	7 (33.3)	14 (66.7)	0.618^a^
70-79	20 (34.5)	38 (65.5)	
80-89	20 (42.6)	27 (57.4)	
≥90 years	11 (47.8)	12 (52.2)	
Sex			
Female	32 (47.1)	36 (52.9)	0.062^a^
Male	26 (32.1)	55 (67.9)	
Marital status			
Single	1 (33.3)	2 (66.7)	0.053^b^
Married	32 (32.7)	66 (67.3)	
Widowed / Divorced	25 (52.1)	23 (47.9)	
Educational level			
Primary	23 (53.5)	20 (46.5)	0.005^a^
Secondary	20 (44.4)	25 (55.6)	
Technical / University	14 (23.3)	46 (76.7)	
Living arrangement			
Lives alone	3 (33.3)	6 (66.7)	0.723^a^
Lives with others	55 (39.3)	85 (60.7)	
Recent loss			
>2 years	34 (37.4)	57 (62.6)	0.666^b^
1-2 years	6 (33.3)	12 (66.7)	
<1 year	18 (45.0)	22 (55.0)	
Harmful habits			
Tobacco	54 (38.3)	87 (61.7)	0.509^a^
Alcohol	8 (32.0)	17 (68.0)	0.436^b^
Time since HTN diagnosis			
<10 years	15 (31.9)	32 (68.1)	0.075^a^
10-19 years	25 (52.1)	23 (47.9)	
≥20 years	18 (33.3)	36 (66.7)	
Number of antihypertensive drugs			
1	34 (42.0)	47 (58.0)	0.705^a^
2	16 (35.6)	29 (64.4)	
≥3	8 (34.8)	15 (65.2)	
Polypharmacy			
1-2	8 (32.0)	17 (68.0)	0.705^ª^
3-4	23 (41.8)	32 (58.2)	
5 or more	27 (39.1)	42 (60.9)	
Charlson Comorbidity Index			
0-2	1 (11.1)	8 (88.9)	0.050^a^
3-4	22 (33.3)	44 (66.7)	
5 or more	35 (47.3)	39 (52.7)	
Frailty Index			
Non-frail	31(88.6)	4(11.4)	<0.001^b^
Pre-frail	51(58.0)	37(42.0)	
Frail	9(34.6)	17(65.4)	
Barthel Index of functionality			
Independent	21 (32.3)	44 (67.7)	0.213^b^
Mild dependency	35 (43.2)	46 (56.8)	
Moderate dependency	2 (66.7)	1 (33.3)	
Beta-blocker use			
No	41 (38.0)	67 (62.0)	0.696^a^
Yes	17 (41.5)	24 (58.5)	

HTN: hypertension; n: number; %: percentage.

a. Chi-squared test.

In the multivariate analysis (Table 3), BB use did not show a significant association with depressive symptoms in any of the tested models. In the crude model, the prevalence ratio (PR) was 1.09 (95% CI: 0.70-1.69; p=0.693). In adjusted models, which included combinations of sociodemographic and clinical variables, PRs were close to unity (ranging from 1.08 to 1.18), without reaching statistical significance (p>0.44). These findings remained consistent across all four adjusted models, reinforcing the absence of association.

By contrast, several factors were significantly associated with depressive symptoms. Having a technical or university education was associated with a lower probability of depressive symptoms in three adjusted models, with PRs of 0.43, 0.48, and 0.49 (p<0.003), compared with primary education. Frailty showed a strong association: pre-frail and frail patients had higher prevalence of depressive symptoms, with PRs of 3.25 (95% CI: 1.26-8.36; p=0.014) and 5.07 (95% CI: 1.93-13.3; p=0.001), respectively, in model C. Finally, HTN duration of 10-19 years was also associated with a higher prevalence of depressive symptoms in model B, with a PR of 1.68 (95% CI: 1.02-2.74; p=0.039), compared with those diagnosed less than 10 years earlier.

**Table 3:** Crude and adjusted prevalence ratios of beta-blocker use for depressive symptoms in older adults.

Variables	Crude model PR (95% CI)	p-value	Model A PR (95% CI)	p-value	Model B PR (95% CI)	p-value	Model C PR (95% CI)	p-value	Model D PR (95% CI)	p-value
Beta-blocker use										
No	1.00		1.00		1.00		1.00		1.00	
Yes	1.09 (0.70-1.69)	0.693	1.08 (0.70-1.63)	0.706	1.18 (0.77-1.82)	0.446	1.12 (0.74-1.71)	0.573	1.09 (0.71-1.68)	0.680
Sex										
Female	1.00		1.00		1.00		1.00		1.00	
Male	0.68 (0.45-1.02)	0.065	0.68 (0.46-1.02)	0.063	0.94 (0.58-1.52)		1.01 (0.65-1.57)	0.963	0.90 (0.56-1.43)	0.665
Age										
60-69	1.00		1.00							
70-79	1.03 (0.51-2.09)	0.925	1.04 (0.54-2.01)	0.348						
80-89	1.27 (0.64-2.55)	0.489	1.26 (0.66-2.40)	0.415						
≥90 years	1.43 (0.68-3.01)	0.341	1.44 (0.72-2.87)	0.297						
Educational level										
Primary	1.00				1.00		1.00		1.00	
Secondary	0.83 (0.54-1.27)	0.540			0.81 (0.49-1.33)	0.411	0.83 (0.53-1.31)	0.444	0.88 (0.54-1.42)	0.613
Technical / University	0.43 (0.25-0.74)	0.003			0.43 (0.23-0.81)	0.008	0.48 (0.26-0.86)	0.015	0.49 (0.26-0.93)	0.029
Time since HTN diagnosis										
<10 years	1.00				1.00					
10-19 years	1.63 (0.99-2.68)	0.055			1.68 (1.02-2.74)	0.039				
≥20 years	1.04 (0.59-1.83)	0.880			1.07 (0.62-1.87)	0.792				
Frailty Index										
Non-frail	1.00						1.00			
Pre-frail	3.67 (1.41-9.58)	0.008					3.25 (1.26-8.36)	0.014		
Frail	5.72 (2.17-15.0)	< 0.001					5.07 (1.93-13.3)	0.001		
Charlson Comorbidity Index										
0-2	1.00								1.00	
3-4	3.00 (0.46-19.8)	0.253							2.83 (0.47-16.9)	0.253
5 or more	4.25 (0.66-27.6)	0.129							3.51 (0.59-20.9)	0.168

HTN: hypertension. PR: prevalence ratio. CI: confidence interval.

Model A: adjusted for sex and age (Pseudo R²: 0.014). Model B: adjusted for sex, education, and duration of HTN (Pseudo R²: 0.046). Model C: adjusted for sex, education, and frailty (Pseudo R²: 0.082). Model D: adjusted for sex, education, and Charlson comorbidity index (Pseudo R²: 0.043).

## Discussion

In this study, no significant association was found between BB use and the presence of depressive symptoms in older adults with HTN, as assessed with the PHQ-9 questionnaire. This finding was consistent both in the crude model and in models adjusted for sociodemographic and clinical variables. By contrast, statistically significant associations were observed between depressive symptoms and frailty, educational level, and duration of HTN diagnosis; these findings are discussed below.

Several studies have explored factors contributing to the development of depressive symptoms in patients with hypertension, including pharmacological treatment and its effects, with heterogeneous results. Consistent with our findings, Tao *et al.*, in a systematic review of clinical trials and observational studies, reported no association between BB use and depressive symptoms. ^13^ Similarly, Riemer et al. published a systematic review with meta-analysis of 285 studies comparing BB monotherapy with placebo, finding no significantly increased risk of depressive symptoms with this drug class (OR: 0.97, 95% CI: 0.51-1.84). ^20^ Moreover, Kessing *et al.*, in a nationwide Danish cohort including more than 5.4 million participants with 10 years of follow-up, reported a negative association (HR: 0.90, 95% CI: 0.89-0.91), suggesting a neutral or even protective effect. ^21^ These findings support the possibility that BBs may not be consistently associated with the development of depressive symptoms in older adults. In a prospective cohort, Tully et al. observed a slight reduction in depressive symptoms in patients treated with selective serotonin reuptake inhibitors (SSRIs) and calcium channel blockers after 2 years of follow-up; however, this effect was not sustained at 10 years, suggesting that even the emotional benefits associated with some antihypertensive drugs may be transient. ^22^

In contrast, other studies have reported a positive association between BB use and depressive symptoms. This may be explained by differences in drug lipophilicity, as lipophilic BBs more readily cross the blood-brain barrier and may influence neurotransmitters related to mood regulation. ^11^ In a UK cohort with 5 years of follow-up, Boal *et al.* found that patients on BB monotherapy had a higher risk of depression (HR: 2.11, 95% CI: 1.12-3.98), although participants were aged over 40 years. ^12^ A similar finding was reported by Cao *et al.* in a retrospective Chinese cohort, where BB use was associated with a higher risk of depressive symptoms compared with ARBs (HR: 1.37, 95% CI: 1.32-1.43). ^23^ Likewise, Ying Li *et al.* compared different classes of antihypertensives and found an association between BBs and depression in older adults through meta-analysis, reinforcing the need to consider the emotional profile of patients when prescribing these drugs. ^24^ These results were further confirmed by Zhang *et al.* in a meta-analysis including 44 BB-only treatment studies, showing an association with depression (OR: 1.45, 95% CI: 1.26-1.67). ^25^Additionally, some observational studies have suggested a higher frequency of depressive symptoms with the use of multiple antihypertensives, although without consistently specifying drug class or considering key clinical variables.

In our study, frailty emerged as one of the strongest factors associated with depressive symptoms, consistent with previous research demonstrating the close relationship between functional decline, perceived physical vulnerability, and emotional well-being in older adults. Kraut *et al.* have argued that antihypertensive use might have more harms than benefits in frail older adults due to increased susceptibility to falls, dizziness, and polypharmacy, which can exacerbate functional dependence and depressive symptoms. This highlights the need for comprehensive geriatric assessment, including frailty evaluation and other geriatric syndromes, as well as adequate screening for depressive disorders and multidisciplinary management. ^26^ Higher educational attainment was also associated with a lower frequency of depressive symptoms, possibly due to greater cognitive reserve, improved coping strategies, or better access to social and health resources. ^27^ Finally, patients with HTN diagnosed for 10-19 years had a higher likelihood of depressive symptoms, which may reflect the cumulative emotional burden of long-standing chronic disease, its sustained impact, or the subjective perception of progressive deterioration over time. ^28^

To date, studies assessing the relationship between BB use and depressive symptoms have yielded conflicting results. This lack of consensus creates uncertainty for clinicians when selecting antihypertensive treatment, particularly for patients at risk of depressive symptoms or with a prior diagnosis of depression. In our study, the absence of association supports the hypothesis that not all BBs have the same emotional impact and that patient characteristics, including clinical-functional profile, frailty status, and social environment, may modulate this relationship. Discrepancies across studies may be explained by differences in study design, heterogeneity of populations analysed (including ethnicity, age, and sociodemographic characteristics), and consideration of variables such as treatment adherence. ^29^ In the absence of solid evidence on the emotional effects of BBs, other clinically relevant determinants must be considered. In this context, frailty stands out as a variable of special interest and should be actively integrated into geriatric assessment in routine care. Treatment of frail older adults should preferably start with monotherapy, prioritising BBs, and decisions should be individualised according to functional, cognitive, and emotional profile. Our findings reinforce the importance of a comprehensive approach to HTN management in older adults, addressing not only haemodynamic control but also psychosocial and functional conditions that directly affect quality of life.

This study has several strengths, including the use of validated instruments to evaluate variables (PHQ-9, FRAIL, and Barthel Index) and multivariate analysis adjusted for relevant sociodemographic and clinical variables, which helped control for potential confounders. However, some limitations must also be acknowledged. First, the cross-sectional design does not allow causal relationships between main variables to be established. Second, although no refusals were reported (reducing participation bias), part of the study population consisted of active or retired military personnel, which limits generalisability of the findings to other older adult populations with different functional profiles and reduces external validity. Additionally, the use of non-probability sampling may have introduced selection bias, as the sample was limited to those attending clinics who met eligibility criteria. Furthermore, BB type by lipophilicity was not differentiated, nor were data on treatment duration or adherence collected, and potential combined therapy with other drugs that might modify effects was not considered. ^30^ These factors may have influenced the results. 

This study found no statistically significant association between BB use and depressive symptoms in older adults with HTN. Future research should address this relationship using longitudinal designs, stratification by age, and adequate control of clinical and psychosocial variables that may act as confounders. Such approaches would enable a more accurate understanding of the impact of this drug class on the emotional health of older adults.
